# Malaria and the liver: immunological hide-and-seek or subversion of immunity from within?

**DOI:** 10.3389/fmicb.2015.00041

**Published:** 2015-02-18

**Authors:** Patrick Bertolino, David G. Bowen

**Affiliations:** Liver Immunology Group, Centenary Institute and AW Morrow Gastroenterology and Liver Centre, University of Sydney and Royal Prince Alfred HospitalSydney, NSW, Australia

**Keywords:** sporozoite, hepatocytes, LSEC, Kupffer cells, hepatic stellate cells, dendritic cells, tolerance, T cells

## Abstract

During the pre-erythrocytic asymptomatic phase of malarial infection, sporozoites develop transiently inside less than 100 hepatocytes that subsequently release thousands of merozoites. Killing of these hepatocytes by cytotoxic T cells (CTLs) confers protection to subsequent malarial infection, suggesting that this bottleneck phase in the parasite life cycle can be targeted by vaccination. During natural transmission, although some CTLs are generated in the skin draining lymph nodes, they are unable to eliminate the parasite, suggesting that the liver is important for the sporozoite to escape immune surveillance. The contribution of the organ to this process is unclear. Based on the known ability of several hepatic antigen-presenting cells (APCs) to induce primary activation of CD8 T cells and tolerance, malarial antigens presented by both infected hepatocytes and/or hepatic cross-presenting APCs should result in tolerance. However, our latest model predicts that due to the low frequency of infected hepatocytes, some T cells recognizing sporozoite epitopes with high affinity should differentiate into CTLs. In this review, we discuss two possible models to explain why CTLs generated in the liver and skin draining lymph nodes are unable to eliminate the parasite: (1) sporozoites harness the tolerogenic property of the liver; (2) CTLs are not tolerized but fail to detect infected cells due to sparse infection of hepatocytes and the very short liver stage. We propose that while malaria sporozoites might use the ability of the liver to tolerize both naive and effector cells, they have also developed strategies to decrease the probability of encounter between CTLs and infected liver cells. Thus, we predict that to achieve protection, vaccination strategies should aim to boost intrahepatic activation and/or increase the chance of encounter between sporozoite-specific CTLs and infected hepatocytes.

## Introduction

Worldwide, infection by malaria-causing parasites kills nearly one million people every year (Murray et al., 2012), and is one of the most important causes of morbidity globally. Human disease is caused by the five species of the *Plasmodium* genus. Parasites are introduced by the bite of female *Anophelese* mosquitoes, which act as a primary host in the parasite life cycle. The remainder of the cycle continues in the human host. In endemic areas, individuals are repeatedly infected, and co-infections by different species are common. Natural infection does not generally confer protection and therefore the development of effective vaccines is critical.

As the mosquito probes for blood, the sporozoites contained in the mosquito salivary glands are predominantly released into the dermis. Most of the parasites reside in the skin for between 1 and 6 h, while 20% migrate via the lymph directly into the skin draining lymph nodes (LNs) (Sidjanski and Vanderberg, 1997; Amino et al., 2006) where they are thought to induce or modulate the subsequent anti-parasite immune response (Yamauchi et al., 2007; Guilbride et al., 2012). It is thought that most of the sporozoites fail to migrate to the LNs and are cleared at the site of inoculation, while a small proportion randomly finds its way to the nearest blood vessel (Sidjanski and Vanderberg, 1997). After crossing the endothelial barrier, the sporozoites enter the circulation to reach the liver (Mota et al., 2001; Ishino et al., 2004, 2005; Frevert et al., 2005). This organ is critical for the next phase of the parasite life cycle, the pre-erythrocytic stage.

The pre-erythrocytic asymptomatic cycle phase may represent the Achilles heel of the parasite, as it is during this bottleneck phase that infected hepatocytes could be efficiently targeted and eliminated by cytotoxic CD8 T cells (CTLs) (Lau et al., 2014). CD8 T cells and the cytokines IFN-γ and TNF-α have been reported as being critical for sterile protection against pre-erythrocytic parasites inside hepatocytes in both animal models and humans (Schofield et al., 1987; Weiss et al., 1988; Krzych et al., 2000; Overstreet et al., 2008; Good and Doolan, 2010; Obeid et al., 2013). Interestingly, the most promising and efficient vaccine candidates in human clinical trials are based on live genetically attenuated whole parasites that infect the liver but do not progress to the blood stage (Epstein and Richie, 2013). Thus, targeting antigens expressed during the pre-erythrocytic cycle phase holds great hope and promise for anti-malaria vaccination.

The liver is acknowledged as a site of primary T cell activation that promotes tolerance rather than effective priming (Bertolino et al., 2002; Benseler et al., 2007; Crispe, 2014). Although activation of protective effector T cells specific for one of the major immunodominant sporozoite-derived antigen has been shown to be restricted to skin draining LNs (Chakravarty et al., 2007; Obeid et al., 2013), it is not clear to what extent the liver contributes to the activation of T cells specific for malarial antigens expressed by infected hepatocytes, or whether this activation generates effector T cells. This question is particularly relevant to vaccination strategies that immunize recipients with irradiated sporozoites that infect the liver but arrest their development in hepatocytes without generating blood stage parasites. The high number of irradiated sporozoites used in this type of immunization is several-fold higher than during natural infection by mosquitos, and is necessary to generate sufficient numbers of T cells for protection.

The liver stage of the parasite is often referred to as a silent phase during which the parasite goes under the immunological radar. It is possible that during this phase, the parasite harnesses the ability of the liver to induce tolerance to avoid being eliminated, and thus establishes productive infection. Alternatively, it is possible that CTLs generated in LNs, and potentially liver, fail to detect the very few infected hepatocytes during the short period of the liver phase. This is particularly a limiting factor in rodent models of malaria as the pre-erythrocytic phase lasts for only 2 days. Both models could explain why this phase is asymptomatic, and apparently immunologically silent.

In this review, we will discuss the likelihood that a T cell response is elicited in the liver during the pre-erythrocytic phase, and the predicted outcome arising from such intrahepatic activation. Our arguments support a model in which sporozoites have developed strategies to induce tolerance but also reduce to a minimum the chances of encountering T cells by delaying efficient elimination of infected hepatocytes.

## Sites of T cell priming during the pre-erythrocytic phase

Unless an individual has been previously infected with malaria parasites, their immune repertoire is predominantly naïve with regard to parasite-specific responses. Naïve T cells recirculation is restricted to the blood and lymph. Via these routes, these cells can also access secondary lymphoid tissues (spleen and LNs). In LNs, naive T cells scan for foreign antigens presented by dendritic cells (DCs) in the form of peptide-MHC (p:MHC) complexes. Primary T cell activation by DCs allows blast formation, cytokine secretion, proliferation, and differentiation into cells endowed with effector function and the capacity for trans-endothelial migration and entry into tissue parenchyma.

**Figure 1:**
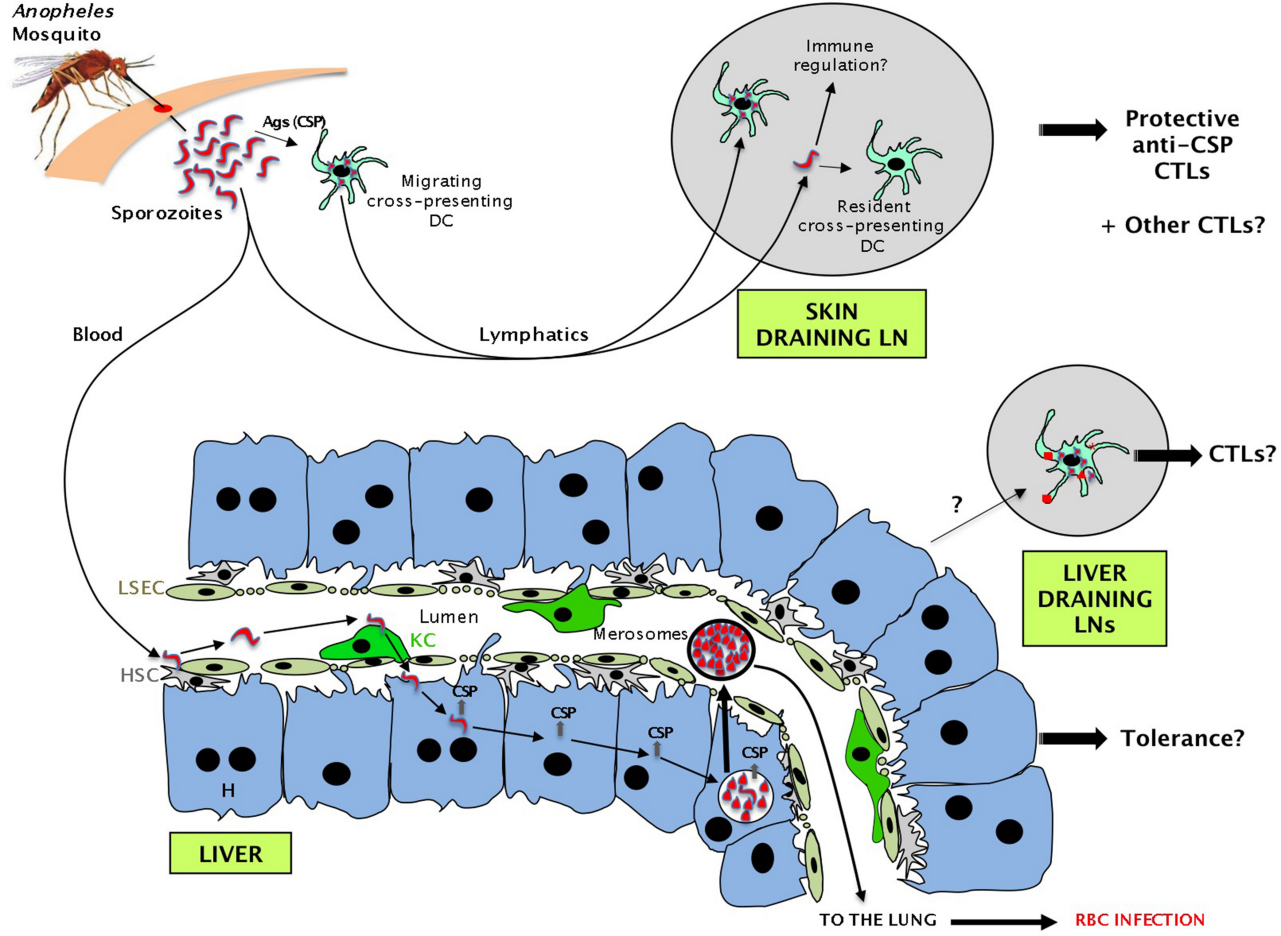
**Sequence of events during the pre-erythrocytic phase of malaria parasite infection leading to the presentation of sporozoite antigens to CD8 T cells in several compartments**. Some sporozoites injected by the mosquito migrate via the lymph into the skin draining lymph nodes (LNs). Migratory DCs or resident DCs capturing sporozoite proteins activate naive CD8 T cells and promote the generation of CTLs. Other sporozoites migrate via the blood to the liver where they are retained by hepatic stellate cells (HSC) and glide. After binding to a Kupffer cell (KC), they cross the liver sinusoidal endothelial cells (LSEC) barrier, traverse through several hepatocytes (H), before entering one in which they establish a parasitophorous vacuole. After a short period (2 days in mice for *P. berghei/P. yoelii* or 7 days in humans for *P. falciparum*), each infected hepatocyte releases thousands of merozoites that will subsequently infect red blood cells. While traversing hepatocytes, sporozoites leave a trail of CSP and possibly other proteins. Antigens shed by infected hepatocytes could also potentially be presented in the liver draining LNs.

As the dermis is the site of inoculation and the first site exposed to sporozoites (Figure 1), it is not surprising that, as assessed by IFN-γ production, the first signs of T cell activation after mouse immunization with irradiated sporozoites (via mosquito bite or intradermal injection) were detected in the skin draining LNs at 48 h (Chakravarty et al., 2007). TCR transgenic T cells specific for the immunodominant antigen, the circumsporozoite protein (CSP), were activated only in these LNs, but effector T cells secreting IFN-γ were not detected in spleen, liver, or liver-draining LNs of the recipients at this time point (Chakravarty et al., 2007). Using a subcutaneous immunization protocol and CD81^−/−^ mice that facilitate restriction of antigen presentation to the skin draining LN rather than the liver, it has been shown that T cell priming in the skin draining LN is sufficient to confer protection (Obeid et al., 2013). Depletion of CD11c^+^ cells abrogated efficient CD8 T cell priming (Jung et al., 2002; Obeid et al., 2013), supporting the model that DCs carrying the sporozoite-derived antigen from the dermis migrated to the draining LNs, where they induced intranodal priming of CSP-specific T cells. Although there is strong evidence that the skin draining LNs prime T cells specific for early pre-erythrocytic proteins such as CSP, other sporozoite proteins are specifically expressed by hepatocytes during the subsequent liver stage. Furthermore, although CSP is an immunodominant antigen in Balb/c mice, it is not immunogenic for CTLs in C57Bl/6 mice, and in most humans naturally infected with malaria parasites (Gruner et al., 2007). Evidence that CSP is not the only protein that contributes to protection includes the development of protective immunity in mice tolerant for CSP that were immunized with irradiated sporozoites (Kumar et al., 2006), and the occurrence of CSP-independent sterile protection under chloroquine prophylaxis after immunization with irradiated sporozoites (Gruner et al., 2007; Mauduit et al., 2010). It is unlikely that the skin-draining LNs play any role in priming CD8 T cells against these mid to late pre-erythrocytic proteins expressed by hepatocytes.

A recent study (Lau et al., 2014) demonstrated that activated TCR transgenic CD8 T cells specific for an unidentified antigen expressed by both liver and blood stage parasites protected against subsequent *Plasmodium* infection, indicating that targeting antigens expressed by hepatocytes can lead to protective immunity. The compartment in which priming occurs is not clear. The liver draining LNs are the most likely site in which CD8 T cells can be primed following cross-presentation of hepatocyte-derived proteins by DCs. However, it is possible that proteins expressed by hepatocytes induce T cell activation and priming in the liver itself.

## The liver: a site of primary activation for CD8 T cells

The liver is now recognized as an alternative site (and the only non-lymphoid organ in the body) in which naive CD8 T cells can be activated independently of lymphoid tissues (Bertolino et al., 2001). First demonstrated for CD8 T cells, this remarkable hepatic immunological property has recently been extended to CD4 T cells (Tay et al., 2014b). Thus, in addition to the hepatic draining LNs, primary activation of T cells specific for mid to late pre-erythrocytic proteins could potentially occur in the liver itself. As proteins such as CSP are also expressed during the early phase of liver infection, intrahepatic tolerance might not only affect naive T cells undergoing primary activation in this organ, but also alter the fate of anti-CSP effector T cells primed in skin draining LNs.

The ability of the liver to support primary CD4 and CD8 T cell activation is likely enabled by its unusual architecture and distinct resident populations of antigen-presenting cells.

### Unique architecture of the liver

The liver receives up to 25% of the cardiac output. Thus, T cells pass through this organ multiple times during their lives, and have the opportunity to interact with hepatic cells located in the sinusoids. The structure of the liver sinusoids is key to understand its role in intrahepatic T cell retention. Liver sinusoids are narrow capillaries with an average diameter of 10 μm, in which blood flow is more intermittent and slower than in other capillaries. This unusual pattern of blood flow is contributed to by the narrow diameter of the sinusoids, the contraction of contractile smooth muscle sphincters in the walls of hepatic arterioles (Macsween and Scothorne, 1979; Macphee et al., 1995), and intermittent obstruction of the lumen by KCs (McCuskey and Reilly, 1993). This unique architecture favors interactions between leukocytes and hepatic cells, and intrahepatic recruitment of T cells recognizing their cognate antigen within this organ. Selectin-dependent leucocyte rolling normally observed in capillaries is not observed in liver sinusoids, and selectins are not required for the recruitment of leucocytes to this organ (Wong et al., 1997; Bowen et al., 2004). In contrast, TCR/p:MHC complex interactions and those between the adhesion molecules LFA-1 and ICAM-1 are important to the retention of lymphocytes, and these have been shown to play a critical role in the recruitment of CD8 T cells to the liver (Bertolino et al., 2005).

### Potential APCs in the liver sinusoids

Several hepatic cell types have been shown to be able to activate naive CD8 T cells *in vitro* (reviewed in Bertolino et al., 2002). It is now accepted that most liver APCs located in the sinusoids are able to induce primary activation *in vivo* as long as they can access circulating T cells and express or capture antigen.

*Hepatocytes* are located behind the endothelial cell layer, and are the principal cell type of the liver, comprising 75% of hepatic cells. These very large polyhedral, mono or bi-nucleated cells contain up to 16N DNA content, and synthesize and secrete a wide variety of biologically crucial molecules. They also metabolize and excrete many endogenous and xenobiotic substances. Steady state hepatocytes express MHC class I molecules (both classical and monomorphic CD1 molecules), and ICAM-1; inflammation also induces expression of MHC class II molecules, CD40L, and costimulatory molecules such as CD80 and CD86 (Bertolino et al., 1998; Holz et al., 2008). Since they normally lack MHC class II expression, resting hepatocytes may therefore only act as APCs for CD1d- and MHC class I-restricted T cells. Primary hepatocytes have also been demonstrated to drive efficient activation and proliferation of naïve TCR transgenic CD8 T cells *in vitro*, confirming their role as efficient APCs (Bertolino et al., 1998).

Despite the widespread view that hepatocytes are in a concealed location, express low levels of MHC class I, and therefore cannot be “seen” by T cells, available data suggests that, despite their sub-endothelial location, this cell population efficiently interacts with circulating CD8 T cells, and functions as effective APCs. *In vivo* interactions between hepatocytes and naïve CD8 T cells have been demonstrated or suggested in different animal models including transgenic mice (Bertolino et al., 2001; Bowen et al., 2004; Derkow et al., 2007), liver transplants (Klein and Crispe, 2006a; Tay et al., 2013), and mice treated with recombinant adeno-associated viral (rAAV) vectors (Wuensch et al., 2006, 2010; Tay et al., 2014a,b). Using electron microscopy, we have shown that circulating T cells make direct contact with hepatocytes through cytoplasmic extensions penetrating the endothelial fenestrations that perforate the LSECs (Warren et al., 2006). Furthermore, in contrast to early data, this study also demonstrated that interactions between T cells and liver parenchymal cells are maximized by the polarized expression of molecules required for primary T cell activation, MHC class I and ICAM-1, on the perisinusoidal cell membrane of hepatocytes (Warren et al., 2006).

*Liver sinusoidal endothelial cells* are efficient scavenger cells strategically located in the sinusoids, and able to clear low-density lipoprotein (LDL) and capture particulate antigens and immune complexes circulating via the blood and deliver them to hepatocytes (Sorensen et al., 2012). This uptake is mediated by mannose receptor, and has been shown to be more efficient than that mediated by KC, suggesting that LSEC specialize in this function. LSEC constitutively express both MHC class I and class II molecules, low levels of CD86, as well as adhesion molecules including ICAM-1, VCAM-1 and dendritic cell-specific intercellular adhesion molecule 3-grabbing non-integrin (DC-SIGN). They have been shown to activate both naïve CD4 and CD8 T cells *in vitro* (Knolle et al., 1999) and *in vivo* (Limmer et al., 2000).

*Kupffer cells (KC)* are the major population of tissue macrophages in the body, and are relatively heterogeneous (Ikarashi et al., 2013). Although some cells with KC-like properties can be reconstituted from bone marrow after lethal irradiation, most KC are derived from the yolk sac early after birth (Jakubzick et al., 2013). Intravital spinning disk and multiphoton microscopy experiments have demonstrated that KC are able to efficiently capture pathogens circulating within the sinusoids, such as *Borrelia burgdorferi* (Lee et al., 2010), and contribute to the formation of hepatic granulomas in *Leishmania donovani* (Beattie et al., 2010) and *Mycobacterium bovis* BCG (Egen et al., 2008) infections. KCs express MHC class I and II molecules, ICAM-1, CD86, CD80 and function as APCs for naïve CD8 T cells and CD4 T cells *in vitro* (Bertolino et al., 2002; Ebrahimkhani et al., 2011). *In vivo*, there is now data showing that liver bone marrow-derived cells (which include both KC and DC) are able to efficiently retain adoptively transferred naïve TCR transgenic CD8 T cells in an antigen-specific manner. This retention leads to the rapid activation of the T cells within the liver (Bowen et al., 2002; Holz et al., 2012; Tay et al., 2014b).

*Hepatic stellate cells (HSC)*, also known as Ito cells, are located between LSEC and hepatocytes. Following liver damage and regeneration, they secrete the extracellular matrix that is critical for tissue repair. However, this process is also responsible for the development of fibrosis. In a non-inflamed liver, these cells play a role in regulating blood flow via constriction to modulate the diameter of the sinusoids (Rockey, 2001). Cultured HSC express MHC Class I and II, CD1d, and low levels of CD80 and CD86 (Vinas et al., 2003; Winau et al., 2007). Contact between HSC and T cells has not been directly observed; however, it is likely that they occur via LSEC fenestrations as demonstrated for hepatocytes (Warren et al., 2006). The role of these cells in intrahepatic T cell activation is still controversial. However, a key study has demonstrated that HSCs are also able to activate naïve CD8 T cells within the liver (Winau et al., 2007). In contrast, a more recent study suggested that HSC are poor T cell stimulators and function more as regulatory bystanders by promoting Tregs and suppressing Th17 differentiation (Ichikawa et al., 2011). These variant results might be due to contaminants in HSC preparations or to phenotypic changes after culture (Vinas et al., 2003). Thus, further *in vivo* data is required to clearly demonstrate the role of HSC as APCs in the intact liver.

It is important to note that the liver also contains DCs and biliary epithelial cells. However, these cell types are located within the portal tracts, behind a non-fenestrated endothelial cell layer, and there is no experimental evidence suggesting that they are able to initiate intrahepatic primary T cell activation (Holz et al., 2010b). In addition, hepatic DCs are mostly immature, suggesting that, like most tissue DCs, these cells induce primary T cell activation in liver draining LNs following maturation and migration triggered by inflammation.

### Cross-presentation in the liver

Antigen cross-presentation is a specialized function restricted to a limited number of APCs. In lymphoid tissues, only some DC subsets can perform this function. In the liver, besides liver resident CD8^+^ DCs, cross-presentation has been clearly demonstrated for LSEC, but the role of other hepatic APCs remains controversial.

The role of LSEC in cross-presenting antigen *in vivo* has been demonstrated in a series of *in vitro* and *in vivo* studies by Knolle and colleagues, clearly establishing these cells as the main cross-presenting cells of the liver. Primary activation of CD8 T cells by LSEC generally promotes tolerance (Limmer et al., 2000) via several mechanisms, including decreased expression of CD80 and CD86 molecules required for efficient priming by secretion of the immunosuppressive molecules PGE2 and IL-10 (Knolle et al., 1998), up-regulation of the inhibitory ligand PD-L1 (Diehl et al., 2008), and interaction with DC leading to loss of IL-12, CD80 and CD86 expression (Schildberg et al., 2008). However, LSEC-activated CD8 T cells appear to survive *in vivo* for prolonged periods, and can be reactivated and rescued from deletion upon secondary encounter with DC (Bottcher et al., 2013).

The role of KC in cross-presenting antigen *in vivo* and the outcome of such activation are more controversial. *In vitro*, KC have been shown to inhibit T cell activation by secreting IL-10 (Knolle et al., 1995), but these cells also increase their expression of MHC class II and their APC function upon TLR3 ligation (Maemura et al., 2005; You et al., 2008). *In vivo*, CD8 T cell activation by KC leads to CD8 T cell apoptosis (Holz et al., 2012), suggesting that these APCs induce tolerance in the absence of inflammation. By comparing the ability of hepatocytes, LSEC, and KC to cross-present cell-associated antigen *in vitro*, Ebrahimkhani and colleagues have recently shown that LSEC are more efficient than KC in cross-presenting antigen (Ebrahimkhani et al., 2011).

Although one study also suggests that HSC can cross-present antigen (Winau et al., 2007) they have also been described as poor APCs and tolerogenic (Eksteen et al., 2009). HSC also appear to promote the expansion of FoxP3^+^ regulatory T cells (Jiang et al., 2008; Ichikawa et al., 2011) due to their high expression levels of retinoic acid and TGF-β (Schwabe et al., 2006).

The role of hepatocytes in cross-presentation remains controversial: although some studies have shown that purified hepatocytes cross-present cell-associated antigen *in vitro* (Ebrahimkhani et al., 2011), other studies (Limmer et al., 2000; Bongfen et al., 2007; Cockburn et al., 2011) suggest the opposite.

## Interaction of sporozoites with liver APCs

After entering via the skin, sporozoites migrate to the liver where they glide along the hepatic sinusoids (Figure 1). The various cell populations located in the hepatic sinusoids are critical for the malaria sporozoites to establish infection in the liver.

HSC proteoglycans protruding through the endothelial fenestration into the sinusoidal lumen have been shown to be critical to the initial retention of circulating sporozoites (Frevert et al., 1993; Pradel et al., 2002). Real time intravital microscopy experiments have revealed that following retention, some sporozoites glide along the sinusoid till they interact with KC (Frevert et al., 2005). These cells express chondroitin and heparan sulfate proteoglycans that promote sporozoite arrest (Pradel et al., 2002). The apical cell pole of the parasite then positions itself against KC and after a pause, the sporozoite traverses the KC, pushes slowly across the sinusoidal cell barrier, and reaches the space of Disse (Figure 1) (Frevert et al., 2005). Whether the sporozoite enters via LSEC fenestrations or through the gap separating two adjacent LSEC has not been resolved, but the results of a recent study suggest that 25% of sporozoites can cross LSEC via the paracellular route (Tavares et al., 2013). Once in the space of Disse, sporozoites seem to increase their velocity and migrate for many minutes through several hepatocytes, before they eventually localize to a final one (Frevert et al., 2005) in which they establish a parasitophorous vacuole (Mota et al., 2001). Hepatocytes are required for the parasite to change its form and multiply. After a period of 7 days in humans for *P. falciparum* or 2 days in mice for *P. berghei/P. yoelii*, each infected liver cell releases 10,000–30,000 merozoites that will subsequently infect red blood cells (Figure 1). This classically marks the end of the silent pre-erythrocytic phase, and the beginning of the symptomatic erythrocytic stage of the infection (Kappe et al., 2003; Frevert, 2004; Heussler et al., 2006).

The gateway model, based on observations made using real time intravital microscopy, suggests that sporozoite migration from the sinusoidal lumen into the space of Disse require KC (Baer et al., 2007). Consistent with the critical role of KC, *P. yoelii* infection of mutant CSF-1-deficient *op/op* mice, that possess 75% less KC (and other macrophages), leads to an 80% decrease in the number of infected hepatocytes (Baer et al., 2007). It is not entirely clear why the parasite would require KC to cross the endothelium, and take the risk of triggering innate immunity, instead of directly crossing the endothelium. Interestingly, avian and reptilian *Plasmodium* species invade and replicate in KC and other macrophages instead of infecting hepatocytes. It has been postulated that during evolution in mammalian hosts the parasite has retained the ability to bind to KC, but gained the property of infecting hepatocytes instead of KC (Baer et al., 2007). Electron micrographs often show KC resting in the lumen of the sinusoids, anchored between two adjacent LSEC. For this reason, it has been proposed that KC act as a portal for sporozoites to cross the endothelial barrier and reach the parenchyma (Baer et al., 2007).

A more recent study using intravital laser spinning-disk confocal microscopy has however demonstrated that, although the majority (78%) of all sporozoite crossing events associated with cell-traversal activity involve KC, 22% involve direct cell traversal of the LSEC without involving a KC (Tavares et al., 2013). KC are thus not an absolutely mandatory gateway for sporozoite crossing of the hepatic sinusoidal barrier. Interestingly, this study also provided evidence that live sporozoites deficient in cell traversal activity were trapped and degraded in KC, while only ~10% of control sporozoites (probably those that have exhausted their CTL capacity or alternatively those that are already dead before they arrive in the liver) displayed similar lasting interactions with KC. Although live sporozoites endowed with cell traversal ability are not trapped and killed by KC, they have been reported to inactivate and eventually kill the traversed macrophage resident cell (Klotz and Frevert, 2008). The authors of this study speculated that the main purpose of sporozoite cell traversal in the liver is to avoid parasite clearance (Tavares et al., 2013).

## Role of the liver in primary T cell activation during the pre-erythrocytic phase

Due to the very low number of hepatocytes infected during natural transmission, and the short duration of the liver phase (between 2 and 7 days depending on the host species and the malaria parasite strain) (Overstreet et al., 2008), the role of the liver in priming CD8 T cells during natural transmission has been difficult to address. Some studies have suggested that the low dose of immunogen transmitted via one mosquito inoculum is not sufficient to induce a clonal burst sufficient to generate an effective T cell response (Hafalla et al., 2002). In addition, *Plasmodium* blood stage infection has been shown to inhibit the maturation and the capacity of DCs to initiate immune responses and suppress protective CD8 T cell responses against the liver stage *in vivo* (Ocana-Morgner et al., 2003). Although it does not necessarily extrapolate to natural *Plasmodium* transmission, immunization with high numbers of irradiated or genetically attenuated sporozoites that are capable of infecting hepatocytes, but do not develop in the liver, excludes any effect of blood stage parasites. This type of immunization allows a better understanding of the liver contribution to CD8 T cell priming and is critical in optimizing vaccine strategies.

Experiments performed using CSP-specific transgenic T cells adoptively transferred into mice immunized with irradiated or non-irradiated sporozoites argued against any significant effective CD8 T cell activation in the liver and liver draining LNs (Chakravarty et al., 2007). However, although this study excluded any contribution of the liver in priming protective anti-CSP CD8 T cells after *Plasmodium* infection, it did not rule out a role for the liver in T cell activation during malaria parasite infection for 2 main reasons. Firstly, T cell activation in this study was assessed by IFN-γ production at 48h instead of other functionally independent markers of T cell activation at earlier time points. Although a hallmark of efficient priming and effector CD8 T cell function, IFN-γ is poorly expressed by hepatocyte-activated CD8 T cells (Holz et al., 2008, 2012). In addition, the majority of T cells activated in the liver undergo rapid deletion, leading to tolerance (Holz et al., 2010a). Secondly, this study focused on CSP, a protein expressed by sporozoites before they invade hepatocytes. Expression of this protein decreases after invasion, while other proteins more specific for the liver stage of the parasite start to be expressed.

The role of the liver in priming T cells specific for mid to late pre-erythrocytic proteins therefore remains to be addressed. Genetically attenuated sporozoites, designed to have arrested development at a late liver stage, induced larger and broader CD8 T cell responses compared to radiation attenuated sporozoite or early-arresting genetically attenuated parasite immunizations (Butler et al., 2011). They also promoted superior protection in inbred and outbred mice (Butler et al., 2011), providing further support for the idea that bona-fide liver stage antigens can prime protective immune responses.

A recent study by Michael Bevan and colleagues illustrates why this question is important for anti-malaria parasite vaccination. By using high-throughput screening and irradiated or attenuated sporozoites, these investigators identified a unique CTL response against an epitope derived from the parasite ribosomal L3 antigen (Murphy et al., 2013), a protein expressed during the liver-stage and in erythrocytes. Surprisingly, these CTLs did not follow the general pattern of anti-CSP CTL responses, as they could be expanded by heterologous prime-boost regimens, but not by multiple sporozoite immunizations. These findings illustrate that CTL responses specific for liver-stage proteins might require different immunization strategies than those specific for antigens expressed by the incoming parasite. Although this study did not clarify the mechanisms responsible for these distinct CTL patterns or whether other liver-stage proteins generate similar patterns, it is tempting to speculate that they are caused by different priming conditions in the liver and LNs.

Several studies have shown that intravenous administration of attenuated sporozoites is superior to other routes in conferring protection (Spitalny and Nussenzw, 1972; Richards, 1977; Chatterjee et al., 1999; Douradinha et al., 2007; Epstein et al., 2011) As sporozoites traveling via the intravenous route reach the liver, these experiments support a major contribution of this organ in priming effector cells. A recent study reporting the results of a clinical trial of an anti-malaria parasite vaccination on 80 volunteers, confirmed that intravenous administration of purified irradiated sporozoites promoted protection, while inoculation of the vaccine via the skin was suboptimally immunogenic and protective (Epstein et al., 2011). A high frequency of sporozoite-specific IFN-γ–producing CD8 T cells in the liver was shown to confer protection in mice (Epstein et al., 2011). Although it does not demonstrate the direct role of hepatic cells in priming effector T cells during natural transmission, this study suggests that targeting the liver route of activation might promote protection.

Presentation of liver-stage sporozoite proteins to antigen-specific CD8 T cells can occur either directly by infected hepatocytes (direct MHC Class I presentation pathway), or indirectly by APCs that capture extracellular sporozoite-derived proteins synthesized by hepatocytes and present them in the context of MHC I molecules (cross-presentation pathway). These two pathways are detailed below.

## Role of infected hepatocytes in primary T cell activation in malaria

Although sporozoites interact with many liver cell types, experiments using bone marrow chimeric mice have demonstrated that radio-resistant cells (most likely to be infected hepatocytes) are likely to be the main cells presenting antigens derived from irradiated sporozoites to effector CD8 T cells in the liver (Chakravarty et al., 2007). Furthermore, it has been shown that while DCs cross-present CSP via endosomes, presentation of CSP by hepatocytes requires expression of the protein in the cytosol rather than in endosomes (Cockburn et al., 2011). CSP presentation by hepatocytes was independent of cross-presentation (Cockburn et al., 2011). Collectively, these results provide strong evidence that hepatocytes are the main liver cells infected by irradiated sporozoites, and able to present cytosolic sporozoite antigens to T cells via the direct MHC class I presentation pathway.

Some evidence also suggests that replication-competent malaria parasite-infected hepatocytes contribute to CD8 T cell priming: (i) elimination of hepatic stage parasites abrogates protection generated by administration of irradiated sporozoites (Scheller and Azad, 1995); and (ii) protection induced by live sporozoites in mice treated with chloroquine was abrogated by primaquine treatment that eliminates the parasite from hepatocytes (Belnoue et al., 2004).

Most studies investigating the early immune events in malaria have used mice immunized with a large dose of irradiated sporozoites, and have focused on the abundant CSP that covers the surface of sporozoites. This protein has multiple functions in several stages of the parasite life cycle. It is thought to be a master regulator of the development of the pre-erythrocytic stages (Singh et al., 2007), and inhibits KC immune defense (Usynin et al., 2007). In infected hepatocytes, CSP traverses the membrane of the parasitophorous vesicle, enters the cytoplasm, and translocates to the nucleus where it downregulates expression of the transcription activation factor NF-κB (Singh et al., 2007). This inhibits host inflammatory immune functions, and promotes the development of the liver stage parasite (Singh et al., 2007). Paradoxically, by entering the cytoplasm, CSP exposes itself to proteasomal degradation and processing by the MHC Class I presentation pathway. CSP has been shown to be an immunodominant protective T cell antigen (Kumar et al., 2006). Mouse primary hepatocytes have also been shown to be able to process *Plasmodium berghei* CSP after live sporozoite infection, and present CSP-derived peptides to specific CD8 T cells *in vitro* in a proteasome-dependent manner (Bongfen et al., 2007). A recent study (Ma et al., 2013) performed using human primary hepatocytes has also confirmed that the direct MHC class I presentation pathway is not markedly affected by live *P. berghei* sporozoites: mRNA expression levels of all major components of the MHC class I processing and presentation machinery were not significantly affected by parasite replication at 24 or 48 h post-infection, while the levels of MHC class I expression were decreased only by 10–20% at the surface of infected host cells as compared to either uninfected or untreated controls. Furthermore, ectopic expression of CSP did not interfere with basal MHC class I expression, or IFN-γ and TNF-α-induced upregulation of MHC class I expression (Ma et al., 2013).

These results therefore imply that CSP translocation to the cytoplasm increases the risk of recognition and elimination of infected hepatocytes following presentation of CSP-peptides by the classical MHC class I presentation pathway. More recently, LISP-2, a protein specifically expressed by mid to late stage parasites, has also been shown to be exported to the cytosol from the parasitophorous vacuole (Orito et al., 2013). If this protein is immunogenic, it also have the potential to be an immune target.

It is thought that the important function of CSP in the parasite development justifies why the parasite exposes itself to the immune system in such a way. An alternative view is that primary activation by hepatocytes leads to tolerance, and therefore this is a low risk strategy for the parasite. Expression of CSP and other liver-stage specific proteins on liver cells might also be critical to tolerize effector CD8 T cells primed in skin-draining LNs.

### Outcome of primary CD8 T cell activation by hepatocytes

Hepatocytes are not “professional” APCs as they do not express known costimulatory molecules such as CD80 and CD86. Although these cells are able to induce primary activation and proliferation of CD8 T cells, it is expected that this activation would not be as efficient as priming induced by mature DCs. Our initial *in vitro* work demonstrated that naive CD8 T cells co-cultured with antigen-expressing hepatocytes were activated, proliferated, and started to acquire cytotoxic activity, but died prematurely by apoptosis (Bertolino et al., 1998, 1999), suggesting that hepatocytes promoted T cell deletion and tolerance.

To investigate the fate of CD8 T cells activated by antigen-expressing hepatocytes *in vivo*, we have used transgenic models and rAAV vectors to restrict antigen or relevant p:MHC expression to these cells *in vivo*. Hepatocyte-activated T cells adoptively transferred into mice expressing the cognate p:MHC complex on all hepatocytes were deleted within the first 4 days post-activation, without developing significant cytotoxic activity (Bertolino et al., 2001; Bowen et al., 2004; Holz et al., 2008; Tay et al., 2014a), suggesting that this activation induced tolerance. In these studies, most CD8 T cells were rapidly deleted within the first few hours post-activation as a result of invading hepatocytes and subsequent degradation inside lysosomal compartments (Benseler et al., 2011). Invasion of a cell into another cell is known as “emperipolesis” (Humble et al., 1956; Overholtzer and Brugge, 2008), and is observed in liver sections in autoimmune and viral hepatitis (Dienes, 1989). To distinguish this process of CD8 T cell deletion within hepatocytes from emperipolesis without destruction of the invading cell, we have termed this process “suicidal emperipolesis” (Benseler et al., 2011).

Some T cells surviving suicidal emperipolesis proliferate, but subsequently die as a result of primary activation in the absence of costimulatory molecules. This “death by neglect” has been shown to be associated with low expression of cytokines and high expression of the pro-apoptotic molecule Bim (Holz et al., 2008).

Although hepatocytes generally induce T cell tolerance *in vivo*, recent studies have suggested that this is not always the case. In some experimental systems, CD8 T cells activated by hepatocytes survived and became full effector cells (Wuensch et al., 2006; Klein and Crispe, 2006b; Derkow et al., 2007). Our results suggest that generation of CTLs by hepatocytes requires high TCR affinity interactions and low frequencies of antigen-expressing hepatocytes; in the absence of these requirements, CD8 T cell tolerance ensues in response to hepatocyte expressed antigen (Tay et al., 2014a).

In malaria parasite infection, the frequency of sporozoite-infected hepatocytes is low. Our recent studies therefore predict that infected hepatocytes should be able to prime at least some high affinity sporozoite antigen-specific T cells and generate full effector cells that contribute to the anti-malaria CTL pool.

## Role of cross-presenting APCs in the anti-malaria response

Unless the parasite has developed strategies to inhibit this process, it is very likely that following entry into hepatocytes, some antigens are shed, captured, and cross-presented by other cell types in lymphoid tissues and/or the liver (Figure 1).

Although liver DCs are not accessible to naive T cells and do not therefore contribute to intrahepatic activation, it is likely that these APCs play a major role in cross-presenting antigens in the liver draining LNs. In studies of transgenic models and using the rAAV system mentioned above, our results suggest that cross-presentation of antigens expressed by hepatocytes leads to efficient T cell priming, resulting in full effector function (Tay et al., 2014a). We have postulated that antigen presentation in lymphoid tissues is the main pathway facilitating the generation of CTLs directed against liver pathogens (Bowen et al., 2004, 2005).

Until recently, the exact location of the liver-draining LNs in mice had not been identified (Barbier et al., 2012). Although likely, the contribution of these lymph nodes to CTL generation during natural malaria parasite infection has not been clearly demonstrated. However, a recent study (Lau et al., 2014) in which mice were intravenously injected with high numbers of irradiated sporozoites demonstrated preferential malarial-antigen specific CD8 T cell activation and proliferation in the 3 hepatic draining LNs (Barbier et al., 2012). The number of intrahepatic CD11c^+^ cells has also been reported to increase in mice injected with irradiated *P. yoelii* sporozoites or from live parasites inoculated by infected mosquitoes (Leiriao et al., 2005b). These potential APCs have been shown to contain intracellular hepatocyte proteins, suggesting that debris of apoptotic hepatocytes were taken up by liver DCs (or recruited circulating DCs or monocytes) (Leiriao et al., 2005b). Whether these cells migrate to the draining LNs and cross-present sporozoite antigens to T cells has not been investigated in this study. Another study (Jobe et al., 2009) used irradiated sporozoites to demonstrate that conventional CD8^+^ DC able to induce the differentiation of naïve CD8 T cells into CD44^hi^CD45RB^lo^CD62L^lo^ effector memory T cells accumulated in the livers of sporozoite-immunized mice, suggesting that this liver DC subset is involved in the induction of liver-stage antigen-specific CD8 T cells.

It is important to emphasize that most of these studies have used high numbers (5 × 10^4^ to 1 × 10^6^) of irradiated parasites injected intravenously, or mice bitten by 50 mosquitoes, that do not reflect natural transmission. Thus, although DCs might capture and cross-present sporozoite proteins from infected hepatocytes in the liver and/or draining LN when high numbers of sporozoites are administered, the role of DCs in cross-presenting sporozoite antigens derived from hepatocytes in the liver draining LNs during natural transmission requires further investigation.

The function and fate of anti-malaria T cells activated by potential cross-presenting hepatic APCs is relatively unknown. Experiments performed using radiation-induced bone marrow chimeras (Chakravarty et al., 2007) excluded any role for bone marrow–derived hematopoietic-lineage liver cells, such as blood-derived DCs or monocyte-derived macrophages, in the presentation of irradiated and live sporozoite-derived antigens to effector CD8 T cells, suggesting that these APCs did not present antigens to T cells. It is important to note that, despite the conclusions of the study, these experiments did not exclude a role for KC, as a large proportion of these liver-resident macrophages are radio-resistant (Kennedy and Abkowitz, 1997; Klein et al., 2007). In addition, the role of radio-resistant LSEC and stellate cells has not been clarified. Thus, in the absence of further experimental evidence, any discussion on this topic remains speculative.

It is clear that the parasite avoids being detected by CTLs primed in the skin draining LNs by changing expression of proteins after settling in hepatocytes. Once in the liver, it is likely that it uses other strategies to avoid being eliminated by CD8 T cells specific for liver stage proteins.

## Could sporozoites harness the tolerogenic property of the liver to subvert intrahepatic immunity and prevent elimination of infected cells?

**Figure 2:**
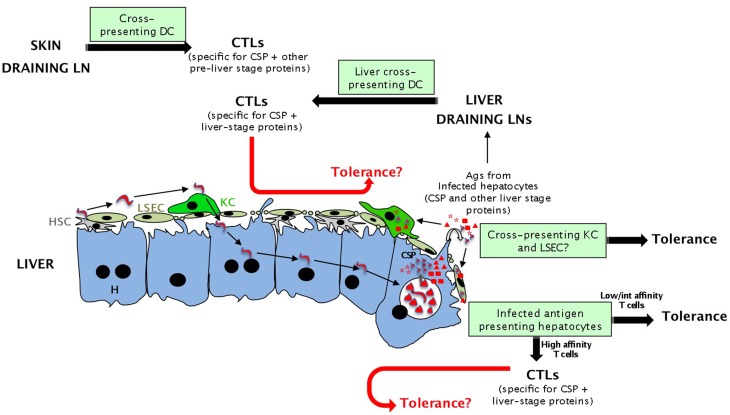
**Potential antigen presenting cells during the pre-erythrocytic phase of malaria parasite infection eliciting tolerance or effective immune responses (CTLs)**. Presentation of sporozoite antigens to CD8 T cells in the liver could occur either by hepatocytes or cross-presenting hepatic cells (LSEC and maybe KC). Although activation by all these cells should lead to tolerance, T cells recognizing sporozoite antigens expressed by infected hepatocytes with high affinity might differentiate into full effector cells. Malarial antigens can also be presented in skin and liver draining LNs by cross-presenting DCs. This activation should lead to efficient priming and CTLs. The fate of effector cells generated in the liver and lymphoid tissues and re-encountering tolerogenic APCs in the liver (red arrows) is unknown.

The first model is that malaria sporozoites prevent the generation of efficient CTLs that would kill infected cells by inducing tolerance (*“subversion from within”* model, see Figure 2). This possibility is more relevant to infection by irradiated sporozoites that do not induce long lasting responses and could be tolerized.

Many studies in the literature suggest that the liver can tolerize effector T cells. In transplantation, an hepatic allograft can reverse rejection of previously transplanted organs from the same donor (Benseler et al., 2007), even when the liver was transplanted up to 6 days after the initial transplant, indicating that the liver allograft is able to induce tolerance in the recently activated T cell compartment. More recently, we have shown that expression of an allo-MHC class I molecule in most hepatocytes prevented the rejection of skin grafts expressing this allo-MHC molecule despite prior sensitization of recipient mice (Cunningham et al., 2013). Finally, analyses performed in transgenic mouse models indicated that TCR transgenic T cells activated in the LNs of recipient mice accumulated in the antigen-expressing liver, killed some hepatocytes, but never induced chronic liver damage, again suggesting that these effector T cells were silenced (Holz et al., 2010b, 2012).

How does the liver tolerize effector T cells? The original “graveyard” model proposed by Crispe and colleagues (Huang et al., 1994; Mehal et al., 1999) suggested that the liver was a disposal site for T cells activated in lymphoid tissues. This was consistent with several studies showing efficient intrahepatic retention of activated CD8 and CD4 T cells. The same group later proposed that the liver actively killed effector T cells recognizing their antigen within the liver. However, as this theory could not account for the presence of effective immune responses in the liver under some circumstances, and did not allow for the presence of high numbers of effector/memory T cells in this organ (Belz et al., 1998; Keating et al., 2007), this concept was subsequently abandoned (Crispe et al., 2006; Bertolino et al., 2007).

We have recently revisited this question by showing that the frequency of antigen-expressing hepatocytes is critical in determining tolerogenic outcome. When at least 25% of hepatocytes expressed antigen, effector T cells primed in lymphoid tissues were eventually silenced, while below this threshold cytotoxic T cells survived after clearing antigen-expressing hepatocytes (Tay et al., 2014a). This might explain the outcome of tolerance in transplantation and transgenic systems, where there is ubiquitous expression of antigen within the liver, and detection of memory T cells in some viral models (Belz et al., 1998; Keating et al., 2007).

The mechanisms by which the liver silences effector CD8 T cells in the presence of high dose antigen is not entirely clear, and molecules such as Galectin-1, TNFR, and FasL on hepatic cells have been suggested to play a key role in this process (reviewed in Holz et al., 2010b, 2012). Our results suggest that most effector T cells surviving the early phases of activation expressed high levels of the pro-apoptotic molecule Bim, and died by apoptosis. T cells that survived this phase displayed poor CTL function, associated with an exhausted phenotype, including expression of high levels of the inhibitory molecule PD-1, as well as reduced TCR and CD3 expression (Holz et al., 2010b, 2012; Tay et al., 2014a), indicative of prolonged antigen exposure and reduced T cell activity (Ferber et al., 1994). It is also possible that suicidal emperipolesis plays an important role in the clearance of effector T cells recognizing their cognate antigen within the liver. This question is currently under investigation in our laboratory.

The extremely low frequency of infected hepatocytes during the pre-erythrocytic stage (even when a high number of irradiated sporozoites is used for immunization) is far from the high antigen dose promoting silencing of effector cells. Based on this argument, we predict that the few infected hepatocytes and potential cross-presenting hepatic APCs should be killed by CTLs, rather than inducing tolerance.

## Do sporozoites avoid being detected by CTLs?

**Figure 3:**
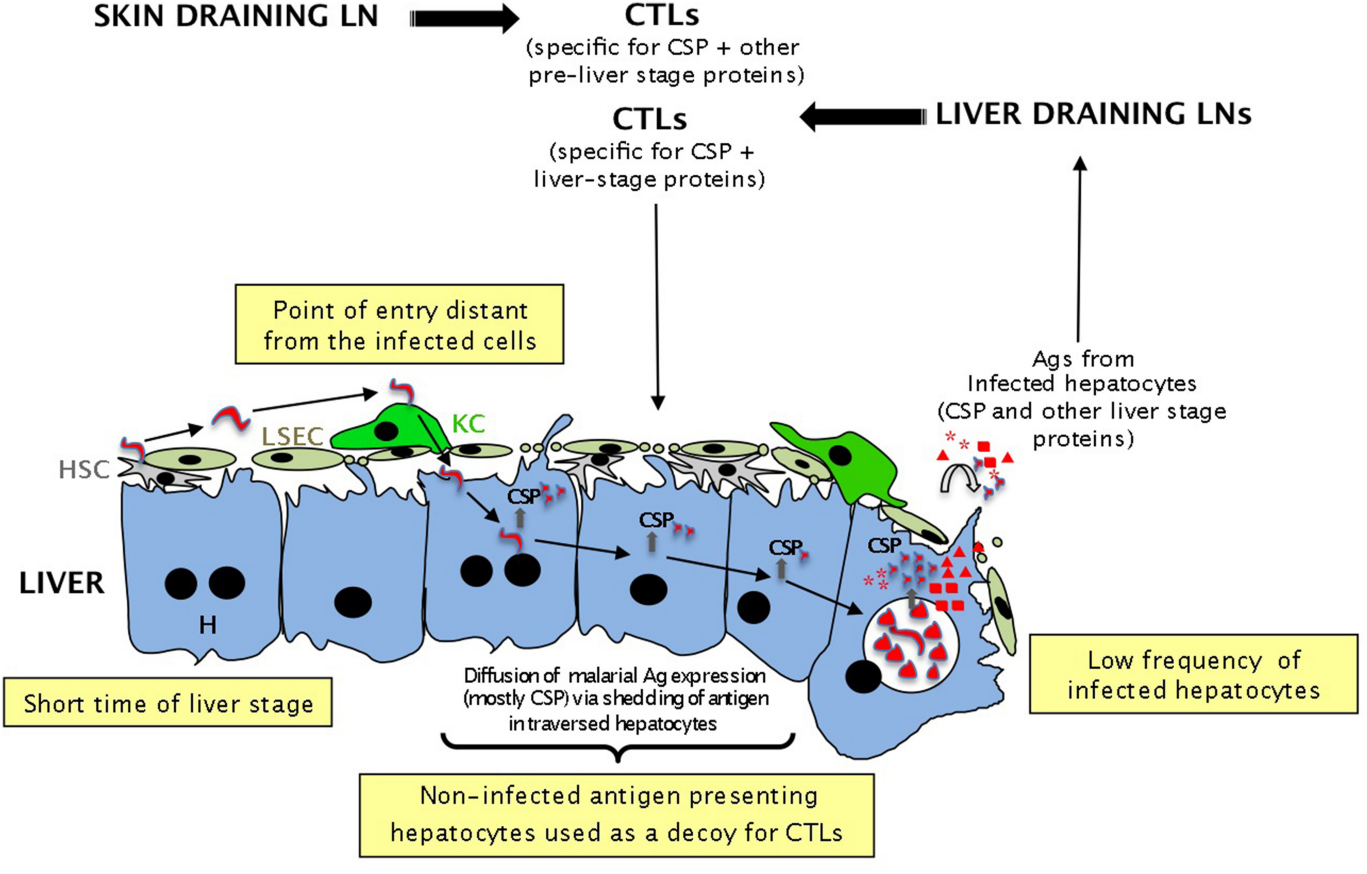
**Different potential strategies used by sporozoites to evade immune surveillance by CTLs according to the “hide and seek” model**. To avoid being eliminated by CTLs generated in the LNs or intrahepatically, malaria sporozoites might have developed several strategies: (i) the very low frequency of sporozoites lowers the probability of encountering CTLs; (ii) the short time spent in the liver (2–7 days) gives CTLs a very short time to kill all infected cells; (iii) infection of hepatocytes at a distance from the entry point might divert the CTLs to the wrong area; (iv) the trail of CSP left in non-infected hepatocytes during cell traversal by sporozoites might be used as a decoy to “buy” some precious time for the sporozoites.

The alternative model to explain how malaria sporozoites use the liver to avoid being eliminated by the immune system is to hide in this large organ to minimize as much as possible the probability of encountering CTLs *(“hide-and-seek” model*, see Figure 3). This model would apply to both live and irradiated sporozoites. From the parasite perspective, it might be efficiently achieved by infecting the minimum number of hepatocytes and by spending the shorter possible time in the host cell.

Using quantitative PCR assays, it has been estimated that a mosquito bite inoculates between 0 and 1297 live sporozoites into the skin, with a mean number of 123 and a median number of 18 (Medica and Sinnis, 2005). As some of these parasites remain in the skin or migrate to the LNs, only a few will make it to the liver (Sidjanski and Vanderberg, 1997; Jin et al., 2007). As a result, an extremely low number of hepatocytes become infected by live sporozoites. It is estimated that in mice, 1 in a million hepatocyte is infected while in humans the frequency is even lower (one in a billion) (Van Braeckel-Budimir and Harty, 2014).

It is not clear whether T cells specific for the liver-stage malaria parasite efficiently find the very few infected hepatocytes during the short period of time they reside in the liver. It is likely that they do not. This is particularly true for naive T cells for primary activation, but it would also be difficult for effector T cells to find their targets (secondary activation). As the liver is made of hundreds of millions of hepatocytes, this is akin to finding a needle in a haystack. Thus, the low number of infected hepatocytes combined with the short amount of time sporozoites reside in the cell might be a strategy used by the parasite to avoid both T cell priming in the liver and escape immune surveillance by CTLs. This might explain the apparent lack of effective immune response during the pre-erythrocytic stage and the lack of long lasting protection induced by both live and irradiated sporozoites. Consistent with this model, high numbers (10^5^) of irradiated sporozoites injected intravenously did not elicit a very significant activation and proliferation of naive TCR transgenic CD8 T cells in the liver during the first 2 days in comparison to the spleen and liver draining LNs, although these T cells recognized an antigen expressed by hepatocytes (Lau et al., 2014). Effector cells generated in lymphoid tissues recirculated, however, to the liver at day 3 (Lau et al., 2014). These findings suggest that, in vaccination protocols using high numbers of irradiated sporozoites, most of the primary T cell activation occurs in lymphoid tissues rather than the liver. Whether this also occurs during natural transmission (lower physiological numbers of live sporozoites inoculated by mosquitos) requires, however, further demonstration. Regardless of whether they are generated in LNs or spleen, to achieve protective immunity CTLs would need to detect and kill all infected hepatocytes during the very short period of the pre-erythrocytic phase (2–7 days). One can predict that the success of this killing is likely to be critically dependent on the total number of effectors.

Results obtained using mouse and human immunization protocols using both attenuated or live sporozoites suggest that this assumption is correct. These protocols can only achieve protection by injecting 1000-fold or more sporozoites than occurs in natural transmission and/or by boosting the number of CSP-specific CD8 T cells (Overstreet et al., 2008). Immunization experiments in mice have suggested that long-term protection requires a remarkably strong CD8 T-cell response, representing a substantial fraction of the total CD8 T-cell pool (Schofield et al., 1987; Weiss et al., 1988; Romero et al., 1989; Schmidt et al., 2008). Optimal CD8 T cell priming was associated with persistence of CSP in both lymphoid tissues and livers of mice, and was mediated by DCs or macrophages (Cockburn et al., 2010). By investigating the CD8 T-cell immune response in Balb/c mice immunized with DC loaded with the immunodominant CSP, followed by a boost provided by *Listeria monocytogenes* expressing CSP, Harty and colleagues have recently shown that stable long term protection upon live sporozoite challenge required CSP-specific CD8 T-cell frequencies exceeding a threshold of 1% of total PBLs (Schmidt et al., 2008). This threshold depends on the mouse MHC strain and *Plasmodium* species (Van Braeckel-Budimir and Harty, 2014). However, it significantly exceeds frequencies of antigen-specific CD8 T cells required for plausible protection against various viral and bacterial infections.

In addition to minimizing the probability of being recognized by T cells whilst within host hepatocytes, it is tempting to speculate that the sporozoite has developed several strategies to gain precious time and further decrease this probability. For rodent malaria parasites (*P. berghei* and *P. yoelii)*, the timing of T cell activation might be sufficient to avoid immune recognition by CTLs activated in LNs: by the time CTLs leave the LNs and circulate to the liver (2–3 days), merozoites would have been generated and the liver phase would be over. The pre-erythrocytic phase of *P. falciparum* in humans is longer (7–9 days) and other strategies might be used by the pathogen to evade or delay immune detection.

One of these potential strategies is to use a decoy to divert the T cells to non-infected cells. When live sporozoites traverse through several hepatocytes before establishing the parasitophorous vacuole in a selected host cell (Frevert et al., 2005), they disrupt the plasma membrane of several other hepatocytes (Mota et al., 2001). During this process, they leave a trail of CSP (and possibly other proteins) in the cytosol of affected cells (Figures 1, 3). It is not clear whether hepatocytes recover from this cell traversal. Studies using high number of live parasites have detected many necrotic hepatocytes and increases in serum ALT (Frevert et al., 2005), suggesting that sporozoites cause hepatocellular injury. Whether this is an experimental artifact due to the high number of parasites, or whether it occurs during natural malaria transmission needs to be clarified. However, if some hepatocytes quickly repair their perforated membranes, it is likely that the CSP contained in the cytosol of non-infected cells will be processed by the MHC class I presentation pathway and form p:MHC complexes. Consistent with this, by using live *P. berghei* sporozoites deficient for the SPECT protein that is essential for cell traversal, Bongfen et al. (2007) have demonstrated that both infected and traversed primary hepatocytes process and present CSP to CD8 T cells. The processing and presentation pathway involved the proteasome, antigen transport through a post-endoplasmic reticulum compartment, and aspartic proteases (Bongfen et al., 2007).

Non-infected hepatocytes presenting CSP might be used by the sporozoite as a decoy strategy to divert CD8 T cells toward non-infected hepatocytes. This tactic might delay the time for T cell detection, and increase the chance of survival of infected hepatocytes. Interestingly, some reports suggested that infected hepatocytes become resistant to apoptosis (Leiriao et al., 2005a; Van De Sand et al., 2005), conferring an additional selective advantage to infected cells.

The reason why the sporozoite needs to traverse several hepatocytes before settling in one cell located at a distant site from the point of entry is also intriguing and could be an additional strategy developed by the parasite to further reduce the risk of being detected by T cells (Figure 3). Upon KC traversal, the abundant CSP on the sporozoite surface has been shown to induce cyclic AMP (cAMP) that inhibits the assembly of the NADPH oxidase (Usynin et al., 2007). This blocks the generation of reactive oxygen species, a potent macrophage defense mechanism required to destroy parasites (Usynin et al., 2007). Although this prevents the parasite from being eliminated by the macrophage defense system while it crosses the endothelium, it is possible that the traversed KC releases cytokines and chemokines able to attract immune cells. This might explain why in addition to blocking the respiratory burst, viable sporozoites also generate an anti-inflammatory cytokine secretion profile in KC (Klotz and Frevert, 2008). KC exposed to infectious sporozoites down-modulated MHC class I, secreted no IL-12p40, and had reduced antigen presenting function (Steers et al., 2005), suggesting that the sporozites minimize the potential signals that KC might release to attract immune cells. Interestingly, in contrast to KC from naive mice exposed to infectious sporozoites, KC from irradiated sporozoite-immune-challenged mice up-regulated class I and costimulatory molecules and produced elevated IL-12p40 relative to naive KC. These KC also exhibited augmented APC activity (Steers et al., 2005).

Settling in the first hepatocyte at the initial entry point would be highly risky for the parasite, as CD8 T cells would rapidly find and kill the infected cell located close to the signaling KC. Traveling to a distant site might buy precious time for the parasite while it differentiates at a distant location.

In summary, malaria sporozoites seem to have developed several strategies to avoid killing of infected hepatocytes by sporozoite-specific CTLs. These include changing expression of proteins within the infected hepatocyte, infecting an organ where primary activation of CD8 T cells is biased toward the induction of tolerance, and developing several tactics to decrease the probability that infected hepatocytes encounter antigen-specific T cells. These arguments support the view that the predominant limitation for malaria-specific T cells is finding/eliminating all infected hepatocytes during the short period of the hepatic cycle phase, rather than overcoming tolerance in the liver.

Thus, although both malaria sporozoites and hepatotropic viruses target the liver, these pathogens seem to use totally different strategies to escape immune recognition. Instead of infecting many hepatocytes in a short period of time and exploiting the tolerogenic properties of the liver, malaria sporozoites infect very few cells, and have developed additional strategies to decrease the probability of being detected by CTLs. This model predicts that to be effective, anti-malaria vaccination protocols should aim to boost the number of CTLs to increase their probability of finding and killing all infected hepatocytes.

### Conflict of interest statement

The authors declare that the research was conducted in the absence of any commercial or financial relationships that could be construed as a potential conflict of interest.

## Abbreviations

APC: Antigen Presenting Cell
CTL: Cytotoxic T Cell
KC: Kupffer Cell
LSEC: Liver Sinusoidal Endothelial Cell
MHC: Major Histocompatibility Complex
TCR: T cell Receptor
CSP: Circumsporozoite Protein
ICAM-1: Intercellular Adhesion Molecule 1
LFA-1: Lymphocyte Function-Associated Antigen 1
LN: Lymph Node
PBL: Peripheral Blood Lymphocytes
HSC: Hepatic Stellate Cells
rAAV: Recombinant Adeno-Associated Virus
DC: Dendritic Cell.
