# Corrigendum: Malaria and the liver: immunological hide-and-seek or subversion of immunity from within?

**DOI:** 10.3389/fmicb.2015.00460

**Published:** 2015-05-13

**Authors:** Patrick Bertolino, David G. Bowen

**Affiliations:** Liver Immunology Group, Centenary Institute and AW Morrow Gastroenterology and Liver Centre, University of Sydney and Royal Prince Alfred HospitalSydney, NSW, Australia

**Keywords:** LSEC, malaria, sporozoites, CD8 T cells, tolerance

In the original article on page 4, there is an error in the following sentence:

“[…] Liver sinusoidal endothelial cells are efficient scavenger cells strategically located in the sinusoids and able to clear low-density lipoprotein (LDL) and capture particulate antigens and immune complexes circulating via the blood and deliver them to hepatocytes (Sorensen et al., 2012). This uptake is mediated by mannose receptor, and has been shown to be more efficient than that mediated by KC, suggesting that LSEC specialize in this function. […]”

The correct sentence is:

“[…] Liver sinusoidal endothelial cells are efficient scavenger cells strategically located in the sinusoids, and able to clear oxidised or acetylated low-density lipoprotein (LDL) and capture small particles (<200 nm) circulating via the blood (Sorensen et al., 2012). This uptake is mediated by several receptors and has been shown to be more efficient than that mediated by KC, suggesting that LSEC specialize in this function. […]”

## Conflict of interest statement

The authors declare that the research was conducted in the absence of any commercial or financial relationships that could be construed as a potential conflict of interest.

## References

[B1] Sorensen K. K. McCourt P. Berg T. Crossley C. Le Couteur D. Wake K.. (2012). The scavenger endothelial cell: a new player in homeostasis and immunity. Am. J. Physiol. Regul. Integr. Comp. Physiol. 303, R1217–R1230. 10.1152/ajpregu.00686.2011 23076875

